# TuYou-County Pediatric Eye (TYPE) study, design issues, baseline demographic characteristics, and implications: Report number 1

**DOI:** 10.1097/MD.0000000000024670

**Published:** 2021-03-12

**Authors:** Xiaoli Zhang, Yajun Yang, Song Zhang, Han Zhang, Litong Yao, Lei Liu, Huixia Li, Xiaoguang Zhang, Shixuan Guo, Lao Qi, Lili Zhou, Jie She, Bin Zhao, Xiaoyan Bian, Guisen Zhang

**Affiliations:** a Department of Cataract, Baotou Chaoju Eye Hospital, Baotou; b China Medical University, Shenyang; c Department of Retina, Inner Mongolia Chaoju Eye Hospital, Hohhot; d Department of Ophthalmology, The First Hospital of China Medical University, Shenyang; e Yuncheng Eye Hospital, Yuncheng, China.

**Keywords:** microvascular changes, ocular diseases, optical coherence tomography angiography, school-based study

## Abstract

To report the rationale, design, and baseline demographic characteristics of TuYou-County Pediatric Eye study, which mainly aimed to determine the retinal microvascular changes with optical coherence tomography angiography (OCTA) and its association with eye abnormalities in school aged children and adolescents at suburban location in Northern China.

TuYou-County Pediatric Eye study was a school-based survey conducted in TuYou-County. Multi-ethnic (Mongol, Han, and Hui) participants will be followed up for 5 years. Standardized ophthalmological examinations include visual acuity, ocular biometry, retinal photography, and OCTA. A questionnaire survey was conducted to collect variables regarding to eye disease such as parental history of eye diseases, near work, outdoor activities, living and eating habits, etc.

After sampling, 687 participants were eligible for investigation, and 20 students did not attend the investigation, living 667 (response rate, 97.1%) students completed questionnaires and all ocular examinations. The average age of all participants was 14.9 ± 5.11.

TYPE study is the first large-scale school-based multi-ethnic survey in suburban site of Northern China. Continuous identification of retinal microvascular changes with eye diseases will provide new insights into the control related diseases in school-age children and adolescents.

## 1 Introduction

Visual impairment (VI) remains the most important disorders cause of handicapping conditions in childhood today.^[1]^ As one of the most common causes of VI in school age children, refractive errors, has become a significant public health issue worldwide, especially in eastern Asia.^[2,3]^ Previous studies have revealed that the incidence of refractive eye disease in China and the global Chinese population is high, and still increased year by year.^[4,5]^ To date, myopia is the eye disease with the highest in prevalence among adolescents, and there are significant racial differences in refractive errors.^[6,7]^ According to recent work, it is estimated that by 2050, myopia prevalence among children and adolescents aged 3 to 19 years would be about 84% and high myopia has seriously threatened Chinese health as well as life.^[8]^ There are some population-based investigations on Children's refractive status at suburban locations in China (western and eastern of China),^[9,10]^ while the epidemic studies of refractive status among school age population are limited and the prevalence and incidence of myopia in children is still an uncertain issue. Therefore, such a wide spread in accurate occurrence and progression of refractive error among suburban Chinese children needs long-term observation.

In addition, fundus diseases are becoming more and more prominent, becoming the most important blinding eye disease of working-age people.^[11,12]^ However, there is little clinical knowledge about the ocular fundus diseases that adolescents may suffer from, and reports on these diseases are limited. Further, description on retinal blood flow by optical coherence tomography angiography (OCTA) and other common ocular abnormalities such as dry eye among Chinese children and adolescents are unclear, thus, there is a need to conduct a survey on these data in order to improve students’ visual quality.

Generally, the TuYou-County Pediatric Eye (TYPE) Study was designed to investigate the retinal microvascular changes with optical coherence tomography angiography (OCTA) and its association with eye diseases among school age children aged 11 to 18 years. Subsequently, TYPE study will also investigate the prevalence, incidence, and the risk factors of refractive error as well as other ocular diseases among school age children and adolescents. These children and adolescents living in suburban areas of TuYou County, Inner Mongolia autonomous region, in Northern China, will be followed-up annually for 5 years. The objective of this article is to summarize the study rationale, design, and demographic characteristics of the TYPE study.

## 2 Methods

### 2.1 Specific aims

The specific aims of the TYPE study included:

1. to describe microvascular characteristics in eyes defined by the OCTA measured as the vessel density;
2. to investigate the association between retinal microvascular changes with eye abnormalities;
3. to determine the prevalence and incidence of refractive errors in multi-ethnic school age children;
4. to investigate the prevalence of other ocular diseases such as amblyopia, strabismus, color blindness, dry eye, and so on;
5. to document the possible cause-specific factors for those ocular diseases so as to provide strategies for intervention.

### 2.2 Study area

TuYou County, located in the central and western part of the Inner Mongolia Autonomous Region (40°N, 110°E) and belongs to a semi-arid mid-temperate continental monsoon climate, with a population of 0.36 million and an area of 2600 km^2^, was identified as the study area due to its demographic and socio-economic characteristics are similar to the national average, and has multi-ethnic population (Mongol, Han, and Hui). The compulsory education system is well executed in TuYouCounty with enrollment rate of 99.6% in middle schools, respectively.

### 2.3 Sampling method and sample size

TYPE study used a stratified cluster sampling method by a coordination meeting which was held by the local city government before the recruitment. Officials from city governments, the health, and the education departments of Tuyou County provide the detail information on demographic and geographic characteristics of Tuyou County. First, 1 district were randomly selected according to the method of random number table from the 9 districts of the county of TuYou. Among the eligible district, the school was defined as the sampling unit. One junior and 1 senior middle school were randomly selected as sample sources. Secondly, the extracted middle schools are stratified from grade 1 to grade 6, and each grade randomly draws 2 classes, and a total of 687 both junior and senior school students form the final sample. The estimate of the sample size is calculated according to formula (1).

(1) $$N={t_{a}}^{2}pq/d^{2}$$

In the formula, N represents the sample size;

*p* represents the expected prevalence of the sample;

*t*_*a*_ represents the *t* value when the degree of freedom is infinite, when *a* = 0.05;

*d* represents the difference between the prevalence of the sample and the population;

q=1−p;

According to the incidence of myopia in Chinese children from a previous cohort study, the cumulative incidence of myopia was ranging from 10.6% to 23.5%.^[13,14]^ Here we set it as 15%. Assuming a design effect of 2.0, a tolerated error of 0.1 times the myopia incidence and loss of follow-up of 10%, the number of samples were 650.

Inclusion criteria:

1. registry in the sampling school;
2. student participants aged 11 to 18 years; and
3. parent or legal guardian agrees childhood to participate in this investigation.

Exclusion criteria:

1. students who did not complete the questionnaire or eye examinations;
2. students who did not hope to attend this survey.

### 2.4 Ethical approval

Our investigation was performed according to the Declaration of Helsinki and approved by the Institutional Review Board (IRB) of Baotou Chaoju Eye Hospital (No. CHAOJU-BT-2020003). Written informed consent was obtained from parents or legal representatives of all participating children.

### 2.5 Questionnaires

The investigation team included 1 Chief Physician, 1 Attending Physician, 2 Residents, 1 Senior Optometrist, and 2 Optometrists. Investigators were trained intensively 2 weeks before investigation. On the day before investigation, the teacher issues a questionnaire to the identified students. The questionnaire named “Ocular Diseases Questionnaire for School age Children and adolescents” includes 2 parts for students and their parents to fill out. The students and their parents completed a questionnaire about the students’ lifestyles that included factors such as time spent on outdoor activities, near-work, and sleeping; and parental history of refractive errors. The time spent outdoors was defined as the sum of outdoor leisure and sports. All the research subjects and their parents fill out the questionnaire independently and submit it to the investigation team on the day of investigation. After review by the investigation team, the unqualified questionnaire (fouling or lack of information, etc.) will be fed back to participants, and then the research subjects will be revised and supplemented until meeting the requirements. For students in lower grades or lack of judgment, their parents fill out the questionnaire instead.

### 2.6 Body check

The height and weight of each participant were measured. Body-mass index (BMI) was calculated using the formula: weight (kg)/square of height (m^2^). The blood pressure and heart rate were measured with an automated sphygmomanometer (Hem-7000; Omron Corporation).

### 2.7 Clinical examination in ophthalmology

The detailed description of clinical examination in ophthalmology within our study was shown in supplementary file. In brief, our ocular check includes vision inspection, computer optometry, eye movement, anterior segment, dry eye and fundus examination, and results on vision and eye conditions were recovered in The TYPE study database. The definitions of ocular conditions were also listed in supplementary file.

### 2.8 Study quality control

Before investigation, a 2-week prior-training was conducted. The whole survey was conducted in school premises during the school days and hours. Pre-investigation was performed on 5% of participants of other sections which were not included in actual investigation. All examinations were required to be performed according to the standard operating procedure, which was supervised by an epidemiologist (GSZ). For the fieldwork, 2 ophthalmologists (YJY, XYB) worked at the field site to ensure that the procedures were followed strictly.

In order to evaluate the reliability of investigators conducting this study procedure, correlation of surveyed data by the investigators was compared to that of the surveyed data by the supervisor. For example, the mean paired difference between investigators and supervisor was 0.01 (95% CI: 0.00–0.02; *P* = .07) for corrected visual acuity and its intra-class correlation was 0.97 (*P* < .001).

### 2.9 Data processing and statistical analysis

The data of questionnaire survey and clinical examination are carefully checked by the well-trained investigators for their accuracy and completeness. For participants with missing data, or misdata, and their teachers were contacted again for questionnaire survey or ocular examinations. All variables are coded, and data collected were independently double-entered using EPI-data v3.1 (EpiData Project and Data Manager) by 2 data entry persons and the analyzed by SPSS version 23.0 (IBM, Chicago). Continuous data were analyzed using the *t* test or one-way analysis of variance (ANOVA), while categorical data using the Chi-Squared test. Multivariable analysis, including the general linear model and linear regression model will be used to assess the significance of associations between a range of exposures and prevalent myopia at baseline. Multiple logistic regression is used to determine adjusted risk ratios. A *P* value less than .05 was considered statistically significant.

## 3 Results

Among the eligible 687 participants, 20 students did not attend the investigation, living 667 (response rate, 97.1%) students completed questionnaires and all ocular examinations. The average age of 667 participants is 14.9 ± 5.11. The demographic characteristics of including participants were shown in Table 1. Furthermore, Table 2 showed other demographic characteristics as the distribution of study population by age. There was no significant statistic on age between boys and girls.

**Table 1:** Demographic characteristics of study participants.

	Overall	Boys	Girls	*P*
Age	14.9 ± 5.11	14.81 ± 6.96	14.99 ± 2.58	.647^∗^
Nationality (%)				
Han	623 (93.40)	296 (47.51)	327 (52.49)	.053^#^
Mongolian	32 (4.80)	11 (34.37)	21 (65.63)	
Hui	11 (1.65)	2 (18.18)	9 (81.82)	
Others	1 (0.15)	1 (100)	0 (0)	
Daily homework, reading or magazines (weekday, %)				
<2 hours	261 (39.13)	143 (54.79)	118 (45.21)	.002^#^
2–5 hours	363 (54.42)	149 (41.05)	214 (58.95)	
>5 hours	43 (6.45)	18 (41.86)	25 (58.14)	
Daily homework, reading or magazines (weekend, %)				
<2 hours	193 (28.94)	110 (56.99)	83 (43.01)	.002^#^
2–5 hours	336 (50.37)	146 (43.45)	190 (56.55)	
>5 hours	138 (20.69)	54 (39.13)	84 (60.87)	
Watch TV, cell phone or touch screen every day (weekday, %)				
<2 hours	476 (71.36)	209 (43.91)	267 (56.09)	.043^#^
2–5 hours	163 (24.44)	83 (50.92)	80 (49.08)	
>5 hours	28 (4.20)	18 (64.29)	10 (35.71)	
Watch TV, cell phone or touch screen every day (weekend, %)				
<2 hours	235 (35.23)	95 (40.43)	140 (59.57)	.046^#^
2–5 hours	321 (48.13)	156 (48.60)	165 (51.40)	
>5 hours	111 (16.64)	59 (53.15)	52 (46.85)	
Daily outdoor exercise time (weekday, %)				
<2 hours	422 (63.27)	182 (43.13)	240 (56.87)	.002^#^
2–5 hours	186 (27.89)	88 (47.31)	98 (52.69)	
>5 hours	59 (8.84)	40 (67.80)	19 (32.20)	
Daily outdoor exercise time (weekend)				
<2 hours	251 (37.63)	89 (35.46)	162 (64.54)	<.001^#^
2–5 hours	297 (44.53)	138 (46.46)	159 (53.54)	
>5 hours	119 (17.84)	83 (69.75)	36 (30.25)	
Sleeping time (school day, h)	7.31 ± 1.13	7.37 ± 1.06	7.25 ± 1.19	.178^∗^
Sleeping time (weekend, h)	8.99 ± 1.43	8.94 ± 1.57	9.03 ± 1.29	.393^∗^
Reading or study time, intermittent overlooking or rest to relax the eyes (Yes, %)	317 (47.60)	143 (45.11)	174 (54.89)	.743^#^
When using eyes, the distance between eyes and books and screen is more than 30 cm (Yes, %)	391 (58.62)	177 (45.27)	214 (54.73)	.407^#^
The distance of watching TV is more than 2 meters (Yes, %)	548 (82.16)	268 (48.91)	280 (51.09)	.006^#^
Frequent consumption of dairy products (%)	334 (50.23)	171 (51.20)	163 (48.80)	.010^#^
Staple food (%)				
Rice	544 (81.68)	251 (46.14)	293 (53.86)	.112^#^
Cooked wheaten food	98 (14.72)	51 (52.04)	47 (47.96)	
Other	24 (3.60)	7 (29.17)	17 (70.83)	
Main course (%)				
Vegetables	381 (57.12)	141 (37.01)	240 (62.99)	<.001^#^
Meat	274 (41.08)	163 (59.49)	111 (40.51)	
Fish	12 (1.80)	6 (50.00)	6 (50.00)	
Meat (%)				
Pork	483 (72.41)	233 (48.24)	250 (51.76)	.002^#^
Beef	47 (7.05)	24 (51.06)	23 (48.94)	
**Mutton**	25 (3.75)	17 (68.00)	8 (32.00)	
Other	112 (16.79)	36 (32.14)	76 (67.86)	
Flavor (%)				
Sweet	399 (59.82)	176 (44.11)	223 (55.89)	.133^#^
Salt	268 (40.18)	134 (50.00)	134 (50.00)	
Do you like fruit (Yes, %)	624 (93.55)	282 (45.19)	342 (54.81)	.011^#^
Do you like wearing glasses or do you think wearing glasses is fun (Yes, %)	71 (10.64)	35 (49.30)	36 (50.70)	.570^#^
Current eye diseases (%)				
Myopia	331 (49.70)	130 (39.27)	201 (60.73)	.001^#^
Hyperopia	11 (1.65)	5 (45.45)	6 (54.55)	
Amblyopia	6 (0.90)	2 (33.33)	4 (66.67)	
Strabismus	8 (1.20)	7 (87.50)	1 (12.50)	
Normal	225 (33.78)	123 (54.67)	102 (45.33)	
Unclear	85 (12.76)	42 (49.41)	43 (50.59)	
Current treatment (%)				
Wearing glasses	299 (44.89)	114 (38.13)	185 (61.87)	<.001^#^
Corneal plastic lens (contact lens)	10 (1.50)	1 (10.00)	9 (90.00)	
Traditional Chinese medicine treatment	1 (0.15)	1 (100.00)	0 (0)	
Untreated	356 (53.45)	193 (54.21)	163 (45.79)	

TV = television.

∗ By Student *t* test.

# By Kruskal–Wallis test.

**Table 2:** Distribution of study population by age.

	Overall	%	Boys	%	Girls	%	*P*^∗^
11 year	3	0.45	2	66.67	1	33.33	.843
12 year	34	5.14	15	44.12	19	55.88	
13 year	151	22.81	68	45.03	83	54.97	
14 year	78	11.78	32	41.03	46	58.97	
15 year	53	8.01	22	41.51	31	58.49	
16 year	129	19.49	69	53.49	60	46.51	
17 year	142	21.45	68	47.89	74	52.11	
18 year	62	9.36	28	45.16	34	54.84	

∗ By Kruskal–Wallis test.

## 4 Discussion

Currently, we reported the rationale, methodology, and demographic characteristics data of refractive errors and other eye conditions in school age children and adolescents cohort in TuYou County. To date, there were 8 main population-based or school-based surveys on eye conditions among children and adolescents (Table 3). At present, China's urbanization process is very fast, and China's urbanization rate reached 60.60% in 2019. However, the reports on suburban school age children's ocular conditions are limited. Unlike many other large-scale studies of children ocular diseases epidemiology, our TYPE study firstly investigated the distribution of ocular conditions among Chinese suburban participants with multi-ethnic, large sample size, and further aims to fill the gap on childhood dry eye and OCTA features using school-based survey. Similar to our TYPE study, the Gobi Desert Children Eye Study was also in the Inner Mongolia, while it located in Desert with extremely arid a condition which is different with TuYou County.

**Table 3:** Observational studies of ocular diseases among children in China.

References	Location	Investigation year	Rural or urban	Study design	Sample size	Age range (years)	Primary outcomes	Other outcomes
Our study	TuYou (Inner Mongolia, northern of China)	2019	Rural + Suburban	School-based		6–18	Myopia	Refractive errors, dry eye, ocular biometry, fundus diseases, OCTA features, strabismus and amblyopia,
Li^[15]^	Anyang (Henan, middle of China)	OctobertoDecember 2011 for grade 7 February to May 2012 for Grade 1	Rural	School-based	Grade 1 (3112), and grade 7 (2363)	Grade 1 (5.7–9.3), and grade 7 (10.0–15.9)	Visualacuity	amblyopia and strabismus, ocular biometry, OCT features, retinal diseases
Xiang^[16]^	Guangzhou (Guangdong, southern of China)	2006	Urban	Population-based	2567	7–15	Myopia	N.A.
Zhao^[14]^	Shunyidistrict (Beijing, northern of China)	1998	Urban	Population-based	4662	5–13	Refractiveerror	N.A.
He^[17]^	Shanghai (eastern of China)	October–November 2016	Urban	School-based	6295	6–9	Myopia	N.A.
Zhu^[18]^	Nanjing (Jiangsu, eastern of China)	2011–2012	Urban	Population-based	5831	3–6	Ocular alignment	Ocular movement and distance visual acuity
Wu^[19]^	Guanxian County and the city of Weihai	N.A.	Rural+Urban	School-based	6364	4–18	Refractive errors and causes of vision loss	Other eye conditions such as IOP, and ocular boimetry
Yang^[20]^	Ejinaqi (western part of the Inner Mongolia, northern-west of China)	N.A.	the Gobi desert	School-based	1911	6–21	Intraocular Pressure	Other eye conditions such as myopia
Pi LH^[9]^	Yongchuan District (Chongqing, western China)	2006–2007	Suburban	Population-based	3469	6–15	Refractive Status	N.A.

IOP = intraocular pressure, N.A. = not applicable, OCT = optical coherence tomography, OCTA = optical coherence tomography angiography.

In 2017, according to data from Inner Mongolia Bureau of Statistics, the disposable income per capita of TuYou County is 24,101 Yuan, which is in the middle of Inner Mongolia (https://tieba.baidu.com/p/5908224943?pv=1&traceid=). This will adjust income level to cover a strong representation.

The major strengths of TYPE study include first focus on the retinal microvascular changes with OCTA and its association with eye abnormalities in school aged children and adolescents, using a randomized sampling at baseline, comprehensive ocular data, high response rate, and standardized protocol of most of the ocular examinations and questionnaires. These advantages made it possible to achieve specific aims of TYPE.

There are still some limitations to the TYPE study. Firstly, some of the variables might be inaccurate according to the self-reported questionnaires from the children and their parents, even though the questionnaires used in the TYPE study were calibrated for Mongolian such as lifestyle. Secondly, there was relative small sample size in present study duo to the difficulty on examination of OCTA among children.

## 5 Conclusion

Generally, data from the TYPE study will give us insight into pediatric retinal microvascular changes with optical coherence tomography angiography (OCTA) and its association with eye diseases among young, ethnic Chinese, and Mongolia schoolchildren in the suburban of China.

## Author contributions

**Conceptualization:** Yajun Yang.

**Data curation:** Xiaoli Zhang.

**Formal analysis:** Song Zhang.

**Funding acquisition:** Jie She.

**Investigation:** Huixia Li, Shixuan Guo, Lao Qi.

**Methodology:** Litong Yao.

**Project administration:** Guisen Gui Zhang.

**Resources:** Lei Liu.

**Supervision:** Han Zhang, Lili Zhou.

**Validation:** Bin Zhao, Xiaoyan Bian.

**Visualization:** Xiaoguang Zhang.

**Writing – original draft:** Guisen Gui Zhang.

**Writing – review & editing:** Guisen Gui Zhang.

## Supplementary Material

Supplemental Digital Content
