# Supplementary material for: TuYou-County Pediatric Eye (TYPE) study, design issues, baseline demographic characteristics, and implications: Report number 1
Source: Medicine (Baltimore). 2021 Mar 12;100(10):e24670. doi: 10.1097/MD.0000000000024670 (PMC7969316; doi:10.1097/MD.0000000000024670)
Supplement: Supplemental Digital Content [file medi-100-e24670-s001.docx]

**Supplemental Digital Content**

**Part 1. Detailed process of clinical examination in Ophthalmology**

Vision test：In a spacious indoor environment, under medium brightness light, apply the indoor light box type "E" international standard logarithmic vision chart (model: standard logarithmic reading type; Guangdong Yuehua Medical Equipment Co., Ltd.) to check the naked eyesight of primary school students. The examiner is 5m away from the visual acuity chart. The suspension height of the visual acuity chart should be such that the 5.0-line visual mark is on the same level as the subject's eyes. In order to avoid the pinhole effect, pay attention to prevent pupils from squinting and peeking with another eye (supervised and corrected by the examiner). Considering the learning effect, the measurement sequence is from the right eye to the left eye. An ophthalmic examination performed by experienced examiners and ophthalmologists included measurement of presenting visual acuity (PVA), uncorrected visual acuity (VA) and best-corrected VA.

Computer automatic optometry: computer optometry adopts automatic refractometer(RM-8900; Japan Topcon Company).Test the right eye first and then the left eye, three times per eye, and record the average result.

Routine eye examination: pen flashlight to evaluate the eye movements of the outer eye；Slit lamp microscopy for ophthalmology (model: SLM-2ER; Chongqing KanghuaRuiming Technology Co., Ltd.) evaluates the anterior segment of the eye and records whether there are abnormalities in the anterior segment of the eyelid, conjunctiva, cornea, etc.; Direct ophthalmoscope (model: YZ11D; Suzhou 66 Vision Technology Co., Ltd.) checks the crystal, vitreous body and fundus, and records various abnormal conditions.

On the day of the examination, if there is a refractive error without wearing a mirror and a patient with eye disease, the result will be recorded in the myopia prevention and control manual. After the questionnaire survey and data collection are completed, it will be distributed to the students and the class teacher will be notified. The class teacher will inform the parents of the students and suggest that they take the children to the local hospital for optician ship for further diagnosis and treatment.
Ocular biometry: Keratometry, central corneal thickness, and white-to-white distance were obtained with a multi-purpose machine (OPD-Scan lllWavefrontAberrometer; Nidek), while the anterior chamber depth and axial length (AL) were measured with an ocular biometer (AL-Scan; Nidek).

Dry eye syndrome (DES): Every participant underwent a dry eye evaluation, which included the Schirmer's test without anesthesia (Schirmer I). [PMID: 32006482]

Intraocular pressure (IOP) was measured in both eyes using the noncontact tonometry (Japan Topcon Company).

Ocular alignment was evaluated by utilizing the Hirschberg light reflex, cover test and prism cover-uncover tests. Cover tests were performed by using fixation targets at both distance (6 m) and near (33 cm).

Fundus camera: Two-field standard, 45-degree, non-mydriatic digital photographic fundus screening were taken of each eye, one centered on the optic disc (field 1) and the other centered on the macula (field 2) using a digital fundus camera (NW7s; Topcon, Tokyo, Japan). In cases where photograph quality was poor, pupillary dilation was conducted if anterior chambers were deemed wide enough to do so safely using the Van Herick method.

Optical coherence tomography angiography (OCTA):OCTA was performed using a commercially available Avanti spectral-domain optical coherence tomography (SD-OCT) device (Optovue, Inc., Fremont, CA, USA) without pupil dilation. The device contained the AngioVue OCTA system (version 2017.1.0.151) and acquired volumetric OCTA scans of the central 6 × 6 mm macula area with a 304 × 304 A-scan sample density at an A-scan rate of 70-kHz and used the split-spectrum amplitude decorrelation angiography (SSADA) algorithm to generate angiograms.

The automatically generated OCTA images were reviewed manually. Scans with a signal strength index (SSI) >50 were included for quantitative analysis. Those with poor scan quality or obvious motion artifacts were excluded. The vessel density of the superficial and deep capillary networks was measured and assessed separately. The superficial retinal capillary network was set from 3 μm below the internal limiting membrane to 15 μm below the inner plexiform layer (IPL) and the deep capillary network was set from 15 to 70 μm below the IPL. The vessel density was defined as the proportion of vessel area with blood flow over the total area measured. Structural SD-OCT images were used for thickness measurement. The inner retinal thickness was measured from the internal limiting membrane (ILM) to the outer boundary of the inner nuclear layer. Full retinal thickness was measured from ILM to the outer boundary of the RPE.

**Part 2.Definition of ocular diseases**

In order to facilitate statistical analysis, this survey unifies the following concepts, definitions and diagnostic criteria of each name:

(1) Visual acuity of the naked eye: distance vision examined by a single eye in its natural state

(2) Equivalent spherical lens (SE): According to the result of one eye refraction, the spherical lens power + 1/2 cylindrical lens power is the equivalent spherical lens power of the eye.

(3) Astigmatism: the absolute value of the diopter of monocular lens is ≥0.50DC or ≥1.00DC. Patients with astigmatism in one or both eyes are astigmatism patients.

(4) Myopia: monocular equivalent spherical power ≤-0.50D or ≤-1.00D. Those with one or both eyes are myopic. Low myopia: -3.00D <SE≤-0.50D; moderate myopia: -6.00D <SE≤-3.00D; high myopia: SE≤-6.00D.

(5) Hyperopia: Monocular equivalent spherical power ≥ + 0.50D or ≥ + 1.00D. A person with hyperopia and another who is not myopic is a hyperopic patient. Low hyperopia: + 0.50D <SE≤ + 3.00D, moderate hyperopia: + 3.00D <SE≤ + 5.00D, high hyperopia: SE> + 5.00D.

(6) Unilateral amblyopia was defined as a 2-line interocular difference in best-corrected VA with ≤20/32 in the worse eye and with coexisting risk factor of 1) strabismus (upon examination or history of strabismus surgery); 2) anisometropia consistent with the eye with worse VA (≥1.00 D SE anisohyperopia, ≥3.00 D SE anisomyopia, or ≥1.50 D anisoastigmatism); or 3) past or present obstruction of the visual axis (e.g. cataract, ptosis, corneal opacity). Bilateral amblyopia was defined as bilaterally decreased best-corrected VA (<20/50 for 30- to 47-month-old or <20/40 for ≥48-month-old) in the presence of bilateral ametropia (≥4.00 D SE hyperopia, ≤-6.00 D SE myopia, or ≥2.50 D astigmatism) or with evidence of visual axis obstruction of both eyes. [PMID: 23697956]

(7) The presence of strabismus, its characteristics (constant or intermittent), type (exotropia, esotropia, hyper/hypotropia, dissociated vertical deviation), A-V strabismus and size (prism diopters) was also recorded. [PMID: 31131243]

(8) DES was diagnosed if there was either the presence of superficial punctate keratopathyon slit lamp examination and/or Schirmer's test results of less than 5 mm.

(9) Any fundus disease was diagnosed if there was either unilateral or bilateral abnormalities in retina or chloride according to photographic or OCTA checks.

**Part 3**: Survey questions and responses in the questionnaire

| **Childhood section**  **Demographics and medical history of child** |
| --- |
| - name, gender, contact information, grade level |
| - Do you have history of premature birth (□no, □yes), or atopic dermatitis (□no, □yes)? |
| - Do you have amblyopia? □no, □yes   - If yes, do you receive any treatment? □no, □yes |
| - Do you have strabismus? □no, □yes   - If yes, do you receive any treatment? □no, □yes |
| - Do you have myopia? □no, □yes - If yes, the refraction status: □less than -3.0D, -3.0D to -5.0D, □-5.0D or higher, □unknown - If yes, do you wear glasses? □no, □yes - If yes, do you receive other treatment (e.g., corneal reshaping contact lens, cycloplegic eye drops)? □no, □yes |
| - What do you prefer to eat? □salty, □sweet, □other - What kinds of food do you prefer to eat? □meat, □eggs, □milk, □vegetables, □others |
| **Near work activities** |
| - What is the distance from the eye to the object when you write, paint, play on the computer, or performs other near work? □≥ 30 cm, □< 30 cm, □unknown |
| - Over the past year, did you use cellphones, computers, or tablet personal computers? □no, □yes - If yes, how old were you when you started to use these products? □< 3 years old, □≥ 3 but <4 years old, □≥ 4 but <5 years old, □≥ 5 but <6 years old □≥ 6 years old - If yes, how much time a day did you spend using these products? □< 1 hour, □≥ 1 but < 2 hours, □≥ 2 but< 4 hours, □≥ 4 hours |
| - Over the past year, how much time a day did you spend watching television? □< 1 hour, □≥ 1 but < 2 hours, □≥ 2 but< 4 hours, □≥ 4 hours |
| - Over the past year, how much time a day did you spend on writing, painting, playing on the computer, or performing other near work? □< 1 hour, □≥ 1 but < 2 hours, □≥ 2 but< 4 hours, □≥ 4 hours |
| **Sleep** |
| - What is the time for you to sleep? □ at 20:00, □ at 21:00, □ at 22:00, □ at 23:00, □ at 23:00, □ at 24:00 or later |
| - What is the time for you to wake up? □ at 6:00 or early, □ at 7:00, □ at 8:00, □ at 8:00 or later |
| **Outdoor activities** |
| - After school, how much time a day on weekdays do you spend on outdoor activities? □none, □< 30 minutes, □≥30 minutes but < 1 hour, □≥ 1 but < 2 hours, □≥ 2 but < 4 hours, □≥ 4 hours |
| - How much time a day on the weekend do you spend on outdoor activities? □none, □< 30 minutes, □≥30 minutes but < 1 hour, □≥ 1 but < 2 hours, □≥ 2 but < 4 hours, □≥ 4 hours |
| **Parents’ information** |
| - What was your child’s feeding style: □breastfeeding, □non- breastfeeding - Education level (father/mother): □illiterate, □primary school, □junior high school, □senior high school/vocational school, □junior college, □university, □graduate school, □unknown |
| - Occupation (father/mother): □no, □work for government/school (soldiers, police, public servants, and teachers), □laborer, □merchant, □self-employment, □agriculture, forestry, fishing, and animal husbandry, □househusband, □others­­_______ |
| - Do you have myopia? (father/mother) □no, □yes, □unknown - If yes, the refraction status is:□less than -5.0D, □-5.0D or higher, □unknown |
| - Do you have any ocular disease? (father/mother) □no, □yes, □unknown - If yes, the disease is___ ____ |
